# The Movement of *Fusarium oxysporum* f.sp. *cubense* (Sub-Tropical Race 4) in Susceptible Cultivars of Banana

**DOI:** 10.3389/fpls.2018.01748

**Published:** 2018-11-27

**Authors:** Noeleen M. Warman, Elizabeth A. B. Aitken

**Affiliations:** ^1^School of Agriculture and Food Sciences, The University of Queensland, St Lucia, QLD, Australia; ^2^Department of Agriculture and Fisheries, Queensland Government, Dutton Park, QLD, Australia

**Keywords:** *Fusarium oxysporum* f.sp. *cubense*, banana, GFP, Fusarium wilt, epidemiology

## Abstract

Fusarium wilt, caused by the fungus *Fusarium oxysporum* f.sp. *cubense* (*Foc*), is one of the most important and destructive diseases in banana crops worldwide. There have been numerous studies into the infection process of this soil-borne pathogen; however, the extent of research into the movement of the pathogen through the rhizome and into the rest of the plant is limited. Furthermore, little is known about the movement of the pathogen once it reaches the aerial components of the plant. A strain of *Foc* sub-tropical race 4, genetically transformed with green fluorescent protein (GFP) gene, was used to monitor the movement of the pathogen through two susceptible cultivars, Cavendish ‘Williams’ (*Musa* AAA) and Lady Finger (*Musa* AAB). Visualization of the pathogen *in planta* demonstrated its presence in the roots, the rhizome and in the outer leaf sheaths of the pseudostem prior to the appearance of external symptoms. Within the non-senescing leaf sheaths, the migration of *Foc* was confined to the xylem vessels; this included those where the vascular tissue was visibly discolored, as well as those, which were apparently healthy. As senescence of leaf sheaths occurred, chlamydospores developed within the gas spaces, while formation of sporodochia, and hyphal growth were apparent on the outer surface of senescing leaf sheaths. These results generate a greater understanding of the epidemiology of *Foc*, providing much needed knowledge to assist in the future management of Fusarium wilt incursions, as well as enhancing protocols for ongoing on-farm hygiene and biosecurity.

## Introduction

Banana (*Musa* spp.) is one of the most important food crops in the world, with many developing countries relying on the fruit as a staple food (Roux et al., 2008; Ploetz, 2015). Banana is grown in approximately 120 tropical and subtropical countries, for both local and export markets (Roux et al., 2008). Up to 40% of global banana production is reliant on the Cavendish subgroup (*Musa* AAA); this includes both domestic markets and the vast majority of export markets. As a consequence of low genetic diversity in banana production pest and disease pressure is one of the major limiting factors worldwide (Ghag et al., 2015). Of these diseases, Fusarium wilt, also known as Panama disease, caused by the fungus *Fusarium oxysporum* f.sp. *cubense* (E.F. Smith) Synder and Hansen (*Foc*), continues to be the greatest threat to global banana production (Ploetz, 2015; Wen et al., 2015). In the mid-20th century, global production of Gros Michel (*Musa* AAA), the former export cultivar of trade, succumbed to Fusarium wilt; the causal agent has since been described as *Foc* race 1 (Stover, 1962; Ploetz, 2005). However the replacement cultivar, Cavendish, is now under threat.

*Fusarium oxysporum* f.sp. *cubense* is classified into four races, based on the host range of cultivars on which they cause disease, but there are at least 24 vegetative compatibility groups (VCG) within the different races (Mostert et al., 2017). The three main races affecting dessert banana include: *Foc* race 1 which causes disease on the cultivar Gros Michel as well as Lady Finger; *Foc* race 2, which affects the same cultivars as race 1 but also the cultivar Bluggoe; and race 4, which causes disease on most cultivars including Cavendish (Ploetz, 2015). Race 3 was at first described as a *Musa* infecting strain but has since been noted to only infect *Heliconia* spp. Initially, race 4 was known only to affect Cavendish cultivars in subtropical areas, where the relatively cooler temperatures are thought to increase Cavendish susceptibility (Ploetz, 2006). However, by the early 1990s, Cavendish cultivars in tropical areas of Southeast Asia began to succumb to Fusarium wilt, and hence, the realization of a “Tropical” race 4 of *Foc* (Ploetz, 2015). Since then different VCGs have been used to distinguish between sub-tropical race 4 (SR4) (VCGs 0120,0129,01211, and 01215) and tropical race 4 (TR4) (VCG 01213-01216) (Fourie et al., 2011). In Australia, Cavendish is the largest grown commercial crop of banana with the majority of production centered in north Queensland^1^. *Foc* TR4 was first identified in Australia in the Northern Territory in 1997 (Condé and Pitkethley, 1999). In 2015, the first ever incident of *Foc* TR4 in Queensland was detected in the Tully region, resulting in regional biosecurity monitoring, and enforcement (O’Neill et al., 2016). With the first wave of Fusarium wilt of banana in the mid-20th century, Stover (1972) then suggested that the only method of controlling the dissemination and subsequent infections by *Foc* in banana was by the quarantine or exclusion of infected properties or by planting non-host crops or cultivars. The same would appear to apply to *Foc* TR4.

Previous research into the infection process of *Foc*, using isolates transformed with the jellyfish green fluorescent pigment (GFP) gene, demonstrated the movement of the pathogen from the soil and into the roots and rhizome (Li et al., 2011, 2017). However, only a limited body of research has been conducted demonstrating the continued movement of the pathogen from the rhizome, through the pseudostem, and into the rest of the plant. Using scanning electron microscopy allowed VanderMolen et al. (1977) to observe the movement of *Foc*, along with the formation of the vascular gels and tyloses, in the xylem vessels. In doing so they found the naturally occurring end walls or perforation plates of the xylem strands somewhat inhibited the movement of the pathogen through the vessels. Microconidia trapped at these plates eventually germinated and were able to penetrate the perforation plate, and so continue to progress through the plant. Beckman (1964) used microscopic red vinyl particles to imitate microconidia and also extrapolated that conidia would be trapped at the scalariform vessel endings. Xiao et al. (2013) used a race 4 isolate of *Foc*, genetically transformed with GFP, to observe the movement through banana plants, tracking it from the roots through to the pseudostem. Using small plants and a large inoculum load, they found that *Foc* traveled through the roots, rhizome, and up the pseudostem, even reaching the outside of the pseudostem, by 24 days post inoculation. At this point the plants were showing severe symptoms or necrosis. Observations of *Foc* in the leaf blades of the plant or development of the long lived chlamydospores were not reported in the study by Xiao et al. (2013).

As a soil borne pathogen, *Foc* persists in the soil on decayed host plant material or as chlamydospores in the absence of a suitable host. Stover (1972) and Ploetz (2006) suggested the chlamydospores are able to remain dormant and viable for up to 30 years in soil. These melanised resting spores germinate in the presence of root exudates from a favorable host. Chlamydospores of other *formae speciales* of *Fusarium oxysporum* have also been reported to cause increased disease severity in their respective crops when compared to microconidia as an inoculum source (Couteaudier and Alabouvette, 1990; Cal et al., 1997).

In the absence of any current commercially viable cultivars resistant to *Foc* TR4 and lack of any effective fungicides, the only means of control is avoidance through quarantine and good hygiene practices. For such, a comprehensive understanding of the epidemiology of *Foc* is necessary. Knowledge of the movement of the pathogen within the plant tissues and its potential to persist in the plant debris is vital to ensure appropriate biosecurity strategies are deployed. The aim of this study was to assess the movement of *Foc* SR4 through entire susceptible banana plants, monitoring its growth in both healthy and senescing sections of the plants. For this purpose, a GFP transformed strain of *Foc* SR4 (*Foc* GFP) was used; quarantine restrictions prohibited the use of *Foc* TR4. Also investigated was the timing and tissue type in which the long-lived chlamydospores were produced.

## Materials and Methods

### Planting Material

Two susceptible banana cultivars were used in this experiment, Cavendish ‘Williams’ (*Musa* AAA) and Lady Finger (*Musa* AAB). Tissue culture plants were deflasked and placed into steam sterilized UQ23 soil mix [70% composted pine bark 0–5 mm, 30% coco peat (coir)] and kept under 12 h fluorescent light for approximately 30 days. Plants were repotted into 140 mm (1.3 L) pots, using steam sterilized UQ23 soil mix and transferred to a containment glasshouse at the St Lucia Campus of The University of Queensland, Brisbane, where they were maintained at 26°C in a natural day light cycle between the months of September to April. For each of the eight time treatments (see below), three replicate plants of each cultivar were prepared as well as three replicates of both cultivars for the controls. Each pot was contained inside a double layer of autoclave bags to contain the genetically transformed *Foc*. To prevent waterlogging, the plants were monitored daily and watered as necessary. Four weeks after repotting, the plants were inoculated as described below. At this stage, the plants had four to five green leaves.

### Inoculation

An isolate of *Foc* SR4 (BRIP 23598, VCG 0120) which had been previously transformed with GFP by Forsyth (2006) was used. The isolate was regenerated on full strength potato dextrose agar (PDA) containing 100 mg L^-1^ of Hygromycin B and incubated for 7 days at 25°C. The millet seed inoculation method was adapted from Smith et al. (2008). Millet seed (*Pennisetum glaucum* (L.) R. Br.) was prepared by soaking overnight, drained, then autoclave sterilized twice at 121°C for 20 min. Following sterilization, each 100 g of the millet was inoculated with a 5 mm round plug of PDA containing *Foc* GFP. The control millet was left uninoculated. The inoculated millet was placed in an incubator set at 25°C for 2 weeks. In preparation for plant inoculation, the millet was ground using a mortar and pestle, and 15 mL of millet was spread around the base of the banana plants and covered with sterile UQ23 mix soil. The plants were not watered until the day after inoculation to allow the *Foc* GFP to colonize. Uninoculated millet was ground and distributed onto the control plants in the same method as for the *Foc* GFP millet.

### Plant Sampling and Confocal Microscopy

**FIGURE 1:**
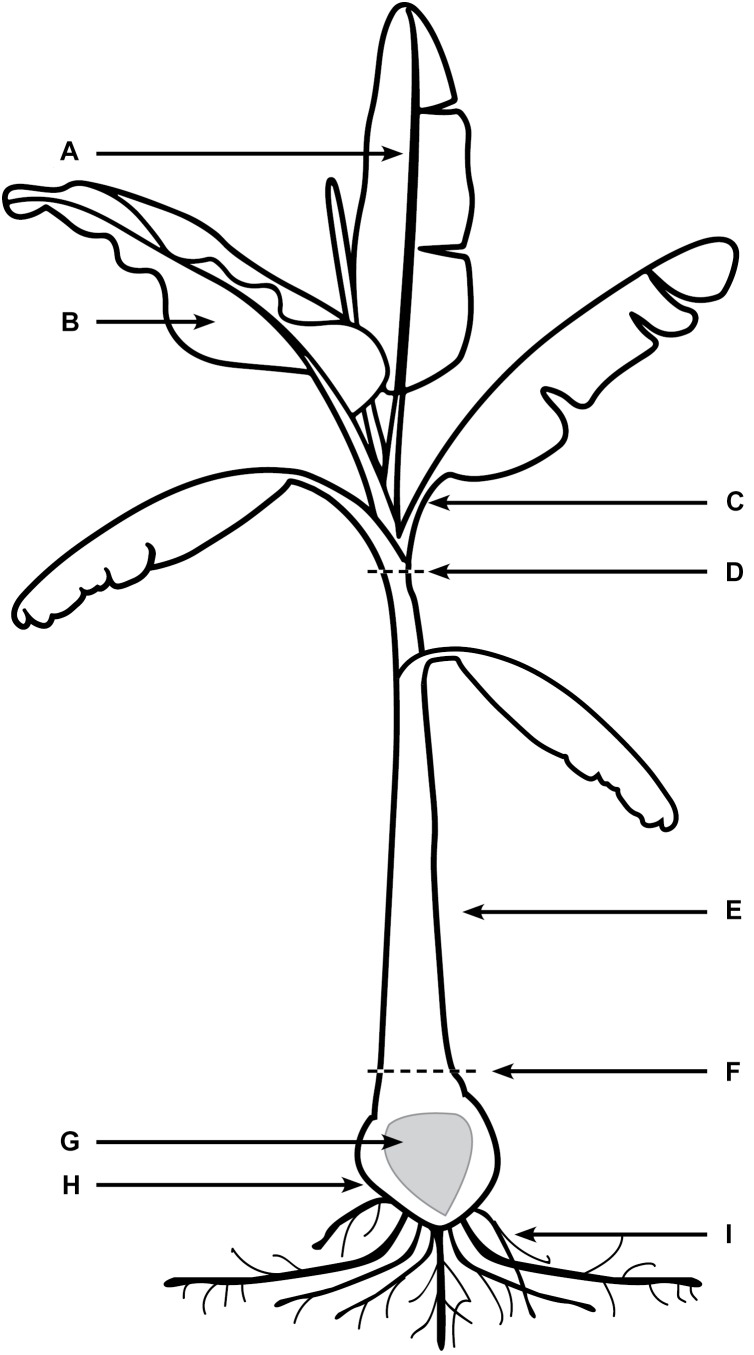
Areas of banana plant used for observation of *Foc* GFP under confocal microscopy. **(A)** Midrib of leaf blade. **(B)** The leaf blade. **(C)** Petiole of the leaf blade. **(D)** Throat of the plant (area of new leaf emergence. **(E)** Leaf sheaths of the pseudostem, including attached senescing sheaths. **(F)** Area of pseudostem emergence above the soil. **(G)** Central cylinder of rhizome. **(H)** Cortex of rhizome. **(I)** Roots including rot tips and sections close to rhizome.

Every 10 days post inoculation (dpi) for a period of 80 days, six Lady Finger and six Cavendish plant were harvested (three controls and three inoculated plants). Individual plants were assessed for external symptoms, which included leaf yellowing, pseudostem splitting, changes in leaf formation such as choking or stunting, petiole collapse, or leaf wilting and skirting of lower necrotic leaves. Each symptom was recorded as 1 for present and 0 for absent. Samples from the entire plant (Figure 1) were observed using confocal microscopy. The pseudostem was segmented into 5 cm pieces from the rhizome upward. These segments included the necrotic outer leaf sheaths as well as the healthy leaf sheaths.

The rhizome and roots were gently shaken to remove loose soil, then rinsed under running water to dislodge any excess soil. Rhizome disease severity ratings were conducted as outlined by Carlier et al. (2003) and described in Table 1.

**Table 1:** Disease severity rating scale for internal symptoms present in rhizome of banana plants caused by *Fusarium oxysporum* f.sp. *cubense* (Carlier et al., 2003).

Rating	Description of symptom
1	Clean rhizome with no evidence of vascular discoloration
2	Isolated points of vascular discoloration
3	Up to 33% of vascular tissue exhibiting discoloration
4	Between 33 and 66% of the vascular tissue discolored
5	Greater than 66% of the vascular tissue discolored
6	Total discoloration of vascular tissue


Transverse and longitudinal sections were hand-sectioned using a double-edged razor blade. Each section was placed onto a microscope slide with drops of water and covered with a glass cover slip. A Zeiss 700 Laser Scanning Microscope was used to perform confocal microscopic examinations equipped with filter blocks with spectral properties matching those of GFP (excitation/emission = 488/555 nm).

### Xylem Fluid Extraction and Examination

Prior to total dissection at each of the time points, inoculated banana plants were dissected in two places; first directly under the throat (Figure 1 zone D), and second, at the soil level (Figure 1 zone F) approximately 2 cm above the rhizome. The initial flow of laticifer sap (latex) was extracted using a 3 mL syringe and placed on a microscope slide with a cover slip. The cut banana tissue was wiped clean using a paper towel sprayed with 70% ethanol and left to sit for 60 min to allow xylem fluid, uncontaminated by latex, to accumulate on the cut surface (David Turner, University of Western Australia and Ken Pegg, Queensland Department of Agriculture and Fisheries Pers.Comm.). After this time, a second extraction was taken and placed onto a microscope slide. The xylem fluid samples were then examined using the confocal fluorescence microscopy to assess for the presence of *Foc* GFP.

## Results

### Disease Development

The first external symptoms of disease occurred in inoculated Cavendish at 20 dpi with the yellowing of the lowest old leaves. By 40 dpi the majority of Cavendish plants were expressing disease symptoms including yellowing of foliage, splitting pseudostem, changes to emerging leaves, wilting foliage and skirting of lower leaves. Whereas these same disease ratings across all inoculated Lady Finger plants occurred at approximately 60 dpi.

**FIGURE 2:**
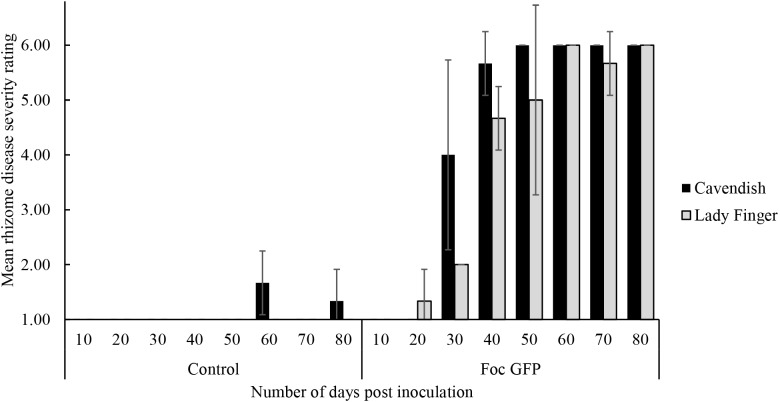
Internal symptoms as mean rating of disease severity, according to the scale in Table 1, in the rhizomes of Cavendish and Lady Finger banana plants recorded in days post inoculation (dpi) with *Foc* GFP infested millet or uninoculated control millet. At each time point, three plants of each treatment were sampled. Data are shown as mean ± standard deviation of the three independent replications (*n* = 3).

With regard to internal symptoms, at 30 dpi, Cavendish plants had developed a mean rhizome disease severity rating of 4, showing 33–66% of rhizome discoloration (Figure 2). At the same time point, Lady Finger rhizomes were displaying only isolated points of slight discoloration. By the end of the assessments (80 dpi), there was no difference in the rhizome disease severity ratings of the Cavendish and Lady Finger cultivars, with all plants displaying 100% rhizome discoloration.

Control plants displaying low level of rhizome discoloration in Figure 2 may be related to potential cross contamination in the glasshouse environment.

### Time Course of Infection as Observed by Confocal Microscopy

**FIGURE 3:**
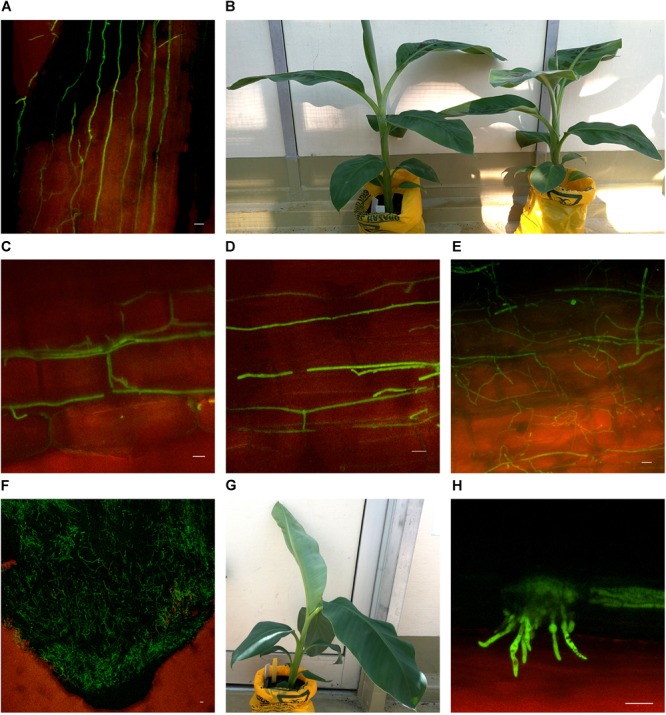
Confocal microscopy highlighting the presence of fluorescing *Fusarium oxysporum* f.sp. *cubense* in different locations in the roots of Cavendish and Lady Finger banana plants. **(A)** Sample from a Cavendish root 20 dpi, showing hyphae apparently progressing through the intercellular spaces in the elongation zone above the root tip. **(B)** Cavendish plants at 20 dpi showing no symptoms of Fusarium wilt, plant on right inoculated with *Foc* GFP and plant on left inoculated with sterile millet. **(C,D)** A Cavendish root 50 dpi, showing hyphae present in the intercellular spaces between cortical cells. **(E)** A sample from a Lady Finger plant at 70 dpi, showing a decaying root with mycelial growth unconfined and throughout the cortical tissue. **(F)** Lady Finger roots 20 dpi, showing a mycelial network covering entire root tip. **(G)** Lady Finger plant at 20 dpi inoculated with *Foc* GFP and not showing symptoms of Fusarium wilt. **(H)** A Cavendish root sample 30 dpi, with macroconidia forming on the outside of root surface. Scale bars represent 20 μm.

Substantial colonization of the roots by *Foc* GFP was observed using the confocal microscope from 10 dpi on both Lady Finger and Cavendish plants. At this time point, all plants were both externally and internally visually symptomless. As time progressed, hyphae were present in the intercellular spaces above the apical meristem of the root tip and in the elongation zone (Figures 3A,C,D). This was also observed when there were no external disease symptoms present in the inoculated plant (Figures 3B,G). Hyphae were also observed in the newly forming xylem vessels in the root zone of maturation. Chlamydospores were noted on the outside of the root tip and among the root hairs. With root tips that showed sign of decay, a hyphal network was apparent covering the entire tip (Figure 3F). In the portions of the senescing roots closest to the rhizome, hyphae were observed intercellularly and were not confined by the xylem (Figure 3E). Macroconidia were also present, appearing attached to the outside of the decaying root tips (Figure 3H) which was observed mainly in the oldest primary roots. In general, the majority of roots assessed showed some level of colonization by the pathogen.

In the inoculated Cavendish plants, the *Foc* GFP appeared to progress internally through the vascular system at a greater rate than that in the Lady Finger plants. At 10 dpi, the fungus was observed to have colonized the roots, rhizome and lower pseudostem of the Cavendish plants, while this did not occur until 20 dpi in the rhizome and 40 dpi in the lower pseudostem of the Lady Finger plants.

**FIGURE 4:**
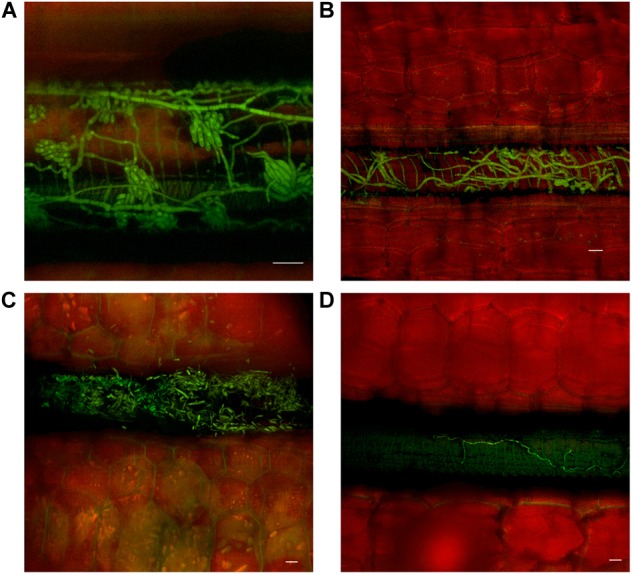
Presence of fluorescing *Fusarium oxysporum* f.sp. *cubense* in the vascular system of the pseudostem of Lady Finger and Cavendish banana plants. **(A)** Lady Finger pseudostem 70 dpi at 10–15 cm above the rhizome showing microconidia formed on the end of monophialides. **(B)** Cavendish pseudostem 80 dpi at 10–15 cm above rhizome showing multiple strands of hyphae confined within a xylem vessel. **(C)** Lady Finger pseudostem 80 dpi at 10–15 cm above rhizome with germinated microconidia apparent within the xylem vessel. **(D)** Lady Finger pseudostem 40 dpi at 10–15 cm above the rhizome showing a single strand of hypha confined to a xylem vessel. Scale bars represent 20 μm.

Movement through the pseudostem occurred first in the outer leaf sheaths and seemed to progressively move from the rhizome to inner sheaths over subsequent time periods. In both cultivars, as vascular discoloration occurred in green leaf sheaths, *Foc* GFP was observed confined to the xylem vessels as single hyphal strands, mycelium, and/or microconidia (Figure 4).

**FIGURE 5:**
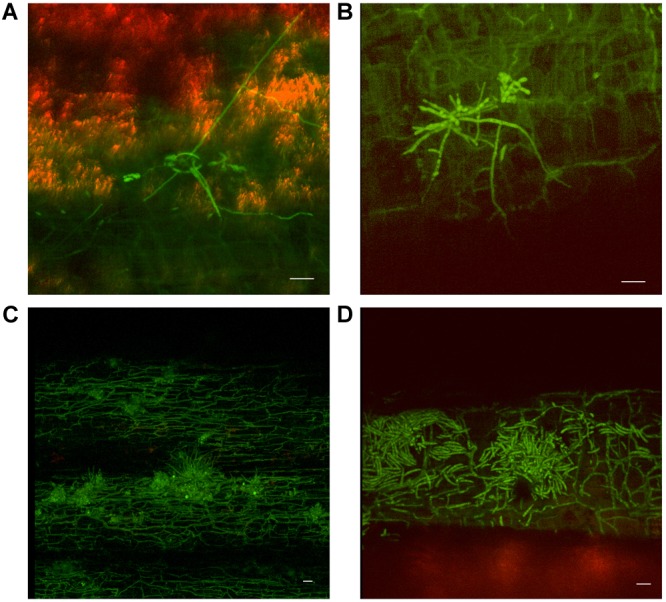
Presence of fluorescing *Fusarium oxysporum* f.sp. *cubense* on the outer surface of dead or senescing leaf sheaths on Cavendish banana plants. **(A)** 80 dpi at 15–20 cm above the rhizome shows hyphae emerging from stoma on the outside of the leaf sheath. **(B)** 70 dpi at 0–5 cm above the rhizome showing hyphae and apparent sporodochia emerging from stoma on the outside of a leaf sheath. **(C)** 50 dpi at 10–15 cm above the rhizome showing mycelium present on the outer leaf sheath surface and sporodochia. **(D)** 40 dpi at 10–15 cm above the rhizome, prolific macroconidia presence on the outer leaf sheath surface. Scale bars represent 20 μm.

As outer leaf sheaths and leaves began to senesce (from 30 dpi to 50 dpi in Cavendish and Lady Finger, respectively), hyphae were observed in the gas spaces of the leaf sheaths and no longer confined to the xylem vessels. At the later stage of disease development, mycelium was observed protruding from stomata in the leaf sheaths and proliferating on the outside of the sheath (Figures 5A,B). At this point, an abundance of macroconidia were arising from apparent sporodochia (Figures 5C,D).

**FIGURE 6:**
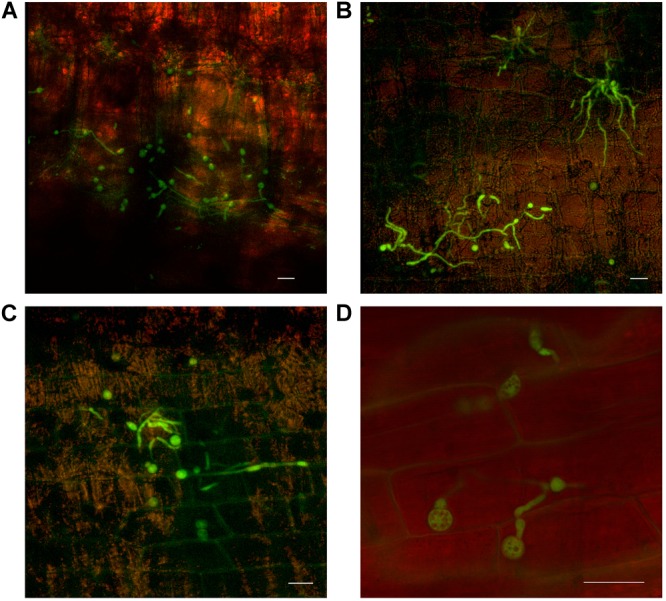
Confocal microscopy highlighting the development of chlamydospores of fluorescing *Fusarium oxysporum* f.sp. *cubense* in decaying leaf sheaths of Lady Finger and Cavendish banana plants. **(A)** Cavendish pseudostem at 40 dpi at 0–5 cm above the rhizome showing chlamydospores present in the gas spaces of leaf sheath. **(B,C)** Cavendish pseudostem at 80 dpi at 5–10 cm above the rhizome showing chlamydospores present on the outside of the leaf sheath. **(D)** Lady Finger pseudostem at 70 dpi at 0–5 cm above rhizome showing the chlamydospores present in decaying leaf sheath. Scale bars represent 20 μm.

Chlamydospore development also occurred, both on the outer surface of the leaf sheath and internally, in the gas spaces (Figure 6). No chlamydospore development was observed in green leaf sheaths of either Lady Finger or Cavendish plants. Additionally, there were no noted chlamydospores on the inside of intact yet discolored xylem vessels.

**FIGURE 7:**
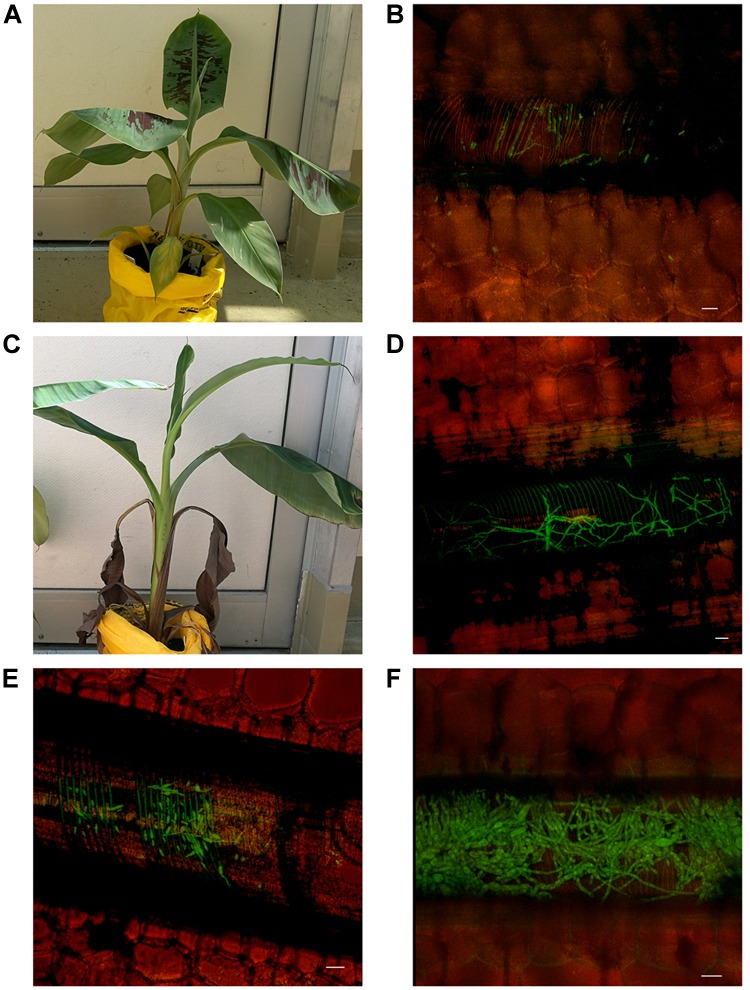
Presence of fluorescing *Fusarium oxysporum* f.sp. *cubense* in the mid rib of leaves above the throat of Lady Finger and Cavendish banana plants. **(A,B)** Cavendish plant 30 dpi inoculated with *Foc* GFP showing limited symptoms of Fusarium wilt with hyphae and microconidia confined to non-discolored xylem vessels of midrib above the throat. **(C,D)** Lady Finger plant 50 dpi inoculated with *Foc* GFP showing hyphae confined to discolored xylem vessel in the midrib of leaf above the throat. **(E)** Cavendish leaf 50 dpi with germinated microconidia confined to xylem vessel. **(F)** Lady Finger leaf 60 dpi, with extensive mycelium appearing to block the xylem vessel. Scale bars represent 20 μm.

By 30 dpi and 50 dpi in Cavendish and Lady Finger plants, respectively, *Foc* GFP was observed in the midrib of the leaves above the throat of the plant. The leaves of the Cavendish (Figure 7A) displayed no chlorosis nor showed any other symptoms typically associated with *Foc*, however, the Lady Finger leaves were distorted (Figure 7C). At this stage the pathogen was confined to the xylem vessels within the midrib (Figures 7B,D). Both mycelium and microconidia were produced continuously in the midrib until the final harvest (80 dpi) (Figures 7E,F). There was no evidence of *Foc* GFP observed on the leaf blades (lamina) throughout the experiment.

### Assessment of Xylem Fluid and Laticifer Sap With *Foc* GFP Inoculated Plants

The xylem fluid and laticifer sap extraction procedures used in this experiment provided only eight positive samples overall from both the Lady Finger and Cavendish banana plants with a total of 84 samples viewed at the different time points (data not shown). Evidence of *Foc* GFP within the sap samples was observed as microconidia and these were observed sporadically within the eight samples. The initial laticifer sap extraction from both the high or low extraction points in the pseudostem were the only samples to provide evidence of *Foc* GFP at a rate of approximately 2–5 conidia per extraction. No *Foc* GFP was observed in the xylem fluid samples. Additionally, several plants failed to produce either xylem fluid or laticifer sap when sections of the pseudostem were removed.

## Discussion

Green fluorescent protein-traced pathogens have been widely used to study infection processes through numerous species and their hosts (Lagopodi et al., 2002; Vallad and Subbarao, 2008; Lü et al., 2014; Yuan et al., 2014). Observing the pathogen which has been transformed with GFP has the distinct advantage of being able to provide a visual analysis of the spore development stages of the pathogen *in planta*. The results of the confocal microscopy in this study provided important information regarding the development of chlamydospores in the pseudostem of the plant, as well as evidence of the pathogen moving through the plant prior to the occurrence of external symptoms. The latter has particular relevance for the management and containment of *Foc* in the field. Similarities between the two cultivars assessed included the confinement of the pathogen to xylem vessels while the leaf sheath was healthy and intact, as well as the movement of the pathogen to the outer surface of decaying leaf sheaths. This is the first known study detailing this progression of *Foc* within entire susceptible Cavendish and Lady Finger banana plants; albeit confined within a glasshouse setting.

The movement of *Foc* through the roots in this study was similar to that previously observed with *Foc* GFP by Li et al. (2011) and Xiao et al. (2013). Chlamydospores and microconidia were observed to germinate around the root tip and among root hairs, prior to penetrating the epidermal cells and moving through the elongation zone intercellularly. However, this study differed from Li et al. (2011) in that no intracellular movement or intracellular reproduction of the pathogen was observed within the roots. These differences may be attributed to the age of the plants when inoculated, banana cultivars assessed, inoculation methods or indeed the use of different fungal isolates; in the current study a sub-tropical race 4 isolate belonging to VCG 120 was used. Intracellular and intercellular movement of the pathogen have, however, been observed using other *formae speciales* of *F. oxysporum* transformed with GFP when assessed on tomato (*Solanum lycopersicum* L.) and strawberry (*Fragaria ananassa* Duch.) (Lagopodi et al., 2002; Yuan et al., 2014).

The various *formae speciales* of *F. oxysporum* are generally regarded as hemi-biotrophs, where the initial infection occurs as a biotroph, and switches to a necrotroph as the plant defense system reacts to the biotrophic invasion (Thaler et al., 2004; Bhadauria et al., 2009). Observations of the movement of the pathogen through the intercellular spaces of the root epidermal cells is characteristic of a biotrophic pathogen and has been noted in the infection process of other *Fusarium* species (Lagopodi et al., 2002; Yuan et al., 2014). Consequently, differences in the potential of strains of *F. oxysporum* to grow inter or intra-cellularly during different stages of the infection process may be highly relevant and worthy of further investigation.

The increasing abundance of *Foc* observed on the outer leaf sheaths as they senesced demonstrates the saprophytic ability of the pathogen to grow continuously on decayed plant material. These decaying outer sheaths were also a location where a high production of chlamydospores was observed. De-leafing is a common practice on banana plantations and is used as a cultural method of controlling banana leaf spot diseases such as black and yellow Sigatoka caused *Pseudocercospora fijiensis* (Morelete) and *Pseudocercospora musae* (Zimm.), respectively (Henderson et al., 2006). This study provides evidence that leaves removed from plants infected with *Foc*, but not yet showing significant symptoms, may later contribute to an increase in the inoculum levels found in the soil. Additionally, the growth of *Foc* on the outside of the leaf sheaths may also provide additional inoculum for aerial dissemination of the pathogen by human or animal/insect influence.

The movement of *Foc* to the outside of the leaf sheath via stomata has seldom been reported. Brandes (1919) noted sporodochia emerging through the stomata on the leaf bases, most commonly at the point where the sheath moves away from the pseudostem. The same development was observed in this study with both sporodochia and mycelia protruding from stomata. A similar study using *Foc* GFP conducted by Xiao et al. (2013) noted *Foc* on the outside of leaf sheaths, however, there was no evidence to show it moving through the stomata. In the study by Xiao et al. (2013), the pathogen was shown to move intercellularly through the decaying leaf sheaths and was observed there 24 days post inoculation. The use of a different isolate, potentially one that was *Foc* TR4, may have resulted in the rapid progression of disease development in their study, resulting in necrotic plants at 24 dpi. Additionally, implementing a bare root dip as the inoculation method may have allowed symptom development and pathogen movement to progress at a faster rate than seen in this current experiment using millet inoculum.

Identifying whether it was microconidia or hyphae that were responsible for the spread of the fungus throughout the pseudostem could not be determined in this study. In some areas, microconidia were observed prior to any signs of hyphae, while at other times, single or multiple strands of hyphae were observed without the presence of any microconidia.

Contrary to observations made by Beckman and Halmos (1962) and Beckman et al. (1962), the pull of microconidia in the transpiration stream and their subsequent trapping in the vessel ends of the vascular elements was not observed. The method of growing the plants axenically and directly inoculating cut roots with a microconidia suspension may have attributed to the observations of Beckman and Halmos (1962) and Beckman et al. (1962), bypassing the infection process undertaken naturally in a soil environment.

The use of *Foc* GFP revealed specific details, highlighting the actual progression and etiology of the pathogen *in planta*. The use of the *Foc* GFP demonstrated the movement of the pathogen from the initial infection of the roots, through the rhizome, into the pseudostem and to the leaves at the top of the plant. Several key findings were observed through this process. The movement of the pathogen to the outer surface of senescing or decayed leaf sheaths, followed by the substantial production of macroconidia and chlamydospores, has serious implications regarding the pathogens potential spread. However how this relates to mature plants in field conditions requires further investigation. Future studies into possible air borne or vectored spread of inoculum and the potential for the pathogen to infect a healthy plant via aerial inoculation are required. Additionally, the production of chlamydospores in and on these outer leaf sheaths increases the risk of long-lived resting spores returning to the soil through cultural methods such as de-leafing. Identification of the progress of the pathogen into the pseudostem prior to external symptom development also provides vital information to assist in the advancement of monitoring and containment protocols currently in place where Fusarium wilt threatens banana production. This study has provided a comprehensive visualization of the movement of the pathogen through banana plants.

## Author Contributions

NW conducted the experimental procedure including glasshouse studies and confocal microscopy; analyzed the results and wrote the majority of the manuscript. EA planned the research, reviewed the results, and edited the manuscript.

## Conflict of Interest Statement

The authors declare that the research was conducted in the absence of any commercial or financial relationships that could be construed as a potential conflict of interest.

## Footnotes

1 https://abgc.org.au/our-industry/key-facts/
